# Risk of SARS-CoV-2 reinfection 18 months after first infection: population-level observational study: Maria Elena Flacco

**DOI:** 10.1093/eurpub/ckac129.284

**Published:** 2022-10-25

**Authors:** ME Flacco, C Acuti Martellucci, G Soldato, G Di Martino, R Carota, A Caponetti, L Manzoli

**Affiliations:** 1 Environmental and Preventive Sciences, University of Ferrara, Ferrara, Italy; 2 Local Health Unit of Pescara, Pescara, Italy; 3 Department of Medical and Surgical Sciences, University of Bologna, Bologna, Italy

## Abstract

**Background:**

Current data suggest that SARS-CoV-2 reinfections are rare. Uncertainties remain, however, on the duration of the natural immunity, its protection against Omicron variant, and on the impact of vaccination to reduce reinfection rates.

**Methods:**

In this retrospective cohort analysis of the entire population of an Italian Region, we followed 1,293,941 subjects from the beginning of the pandemic to the current scenario of Omicron predominance (up to mid-February 2022). We assessed the proportion of reinfections overall, and by demographic and clinical characteristics, time after primary infection, and predominant circulating variant. Cox proportional hazard analysis was used to compute the relative hazards of reinfection.

**Results:**

After an average of 277 days, we recorded 729 reinfections among 119,266 previously infected subjects (overall rate: 6.1‰), eight COVID-19-related hospitalizations (7/100,000), and two deaths. Importantly, the incidence of reinfection did not vary substantially over time: after 18-22 months from the primary infection, the reinfection rate was still 6.7‰, suggesting that protection conferred by natural immunity may last beyond 12 months. The risk of reinfection was significantly higher among females, unvaccinated subjects, and during the Omicron wave.

**Conclusions:**

This study confirms and expands previous findings reporting a low risk of SARS-CoV-2 reinfection, and a very low risk of severe or lethal COVID-19 for those who recovered from primary infection, suggesting that the protection conferred by the natural immunity lasts beyond 12 months. Although the marked increase of the reinfection rates during the Omicron wave is concerning, the risk of a secondary severe disease or death remained close to zero. Vaccines were able to significantly reduce the likelihood of reinfection in both pre-Omicron and Omicron waves, although the risk-benefit profile of multiple vaccine doses for this population should be carefully evaluated.

**Key messages:**

• After primary infection, the risk of SARS-CoV-2 reinfection and of severe/lethal COVID-19 was low, suggesting that natural immunity lasts beyond 12 months.

• Despite increasing reinfection rates with Omicron, the risk of a secondary severe/lethal disease was close to zero, and vaccines reduced the likelihood of reinfection before and during Omicron waves.

